# Supplementary material for: Associations between Brain Alpha-Tocopherol Stereoisomer Profile and Hallmarks of Brain Aging in Centenarians
Source: Antioxidants (Basel). 2024 Aug 17;13(8):997. doi: 10.3390/antiox13080997 (PMC11351880; doi:10.3390/antiox13080997)
Supplement: Supplementary file 1 [file antioxidants-13-00997-s001.zip › Supplemental_Figures.pdf]

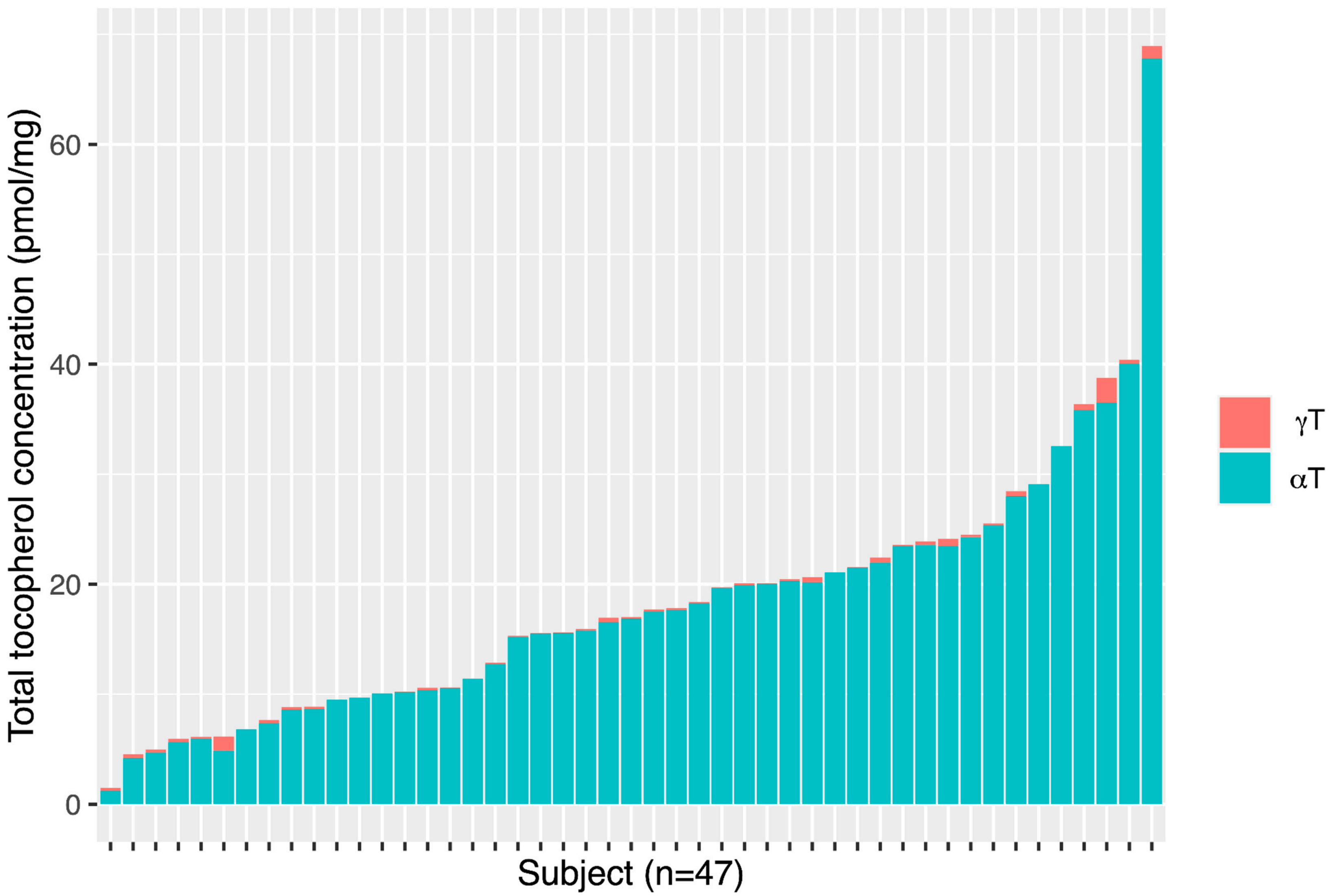

Total tocopherol concentration (pmol/mg)
0
20
40
60
Subject (n=47)
γT
αT

(A)

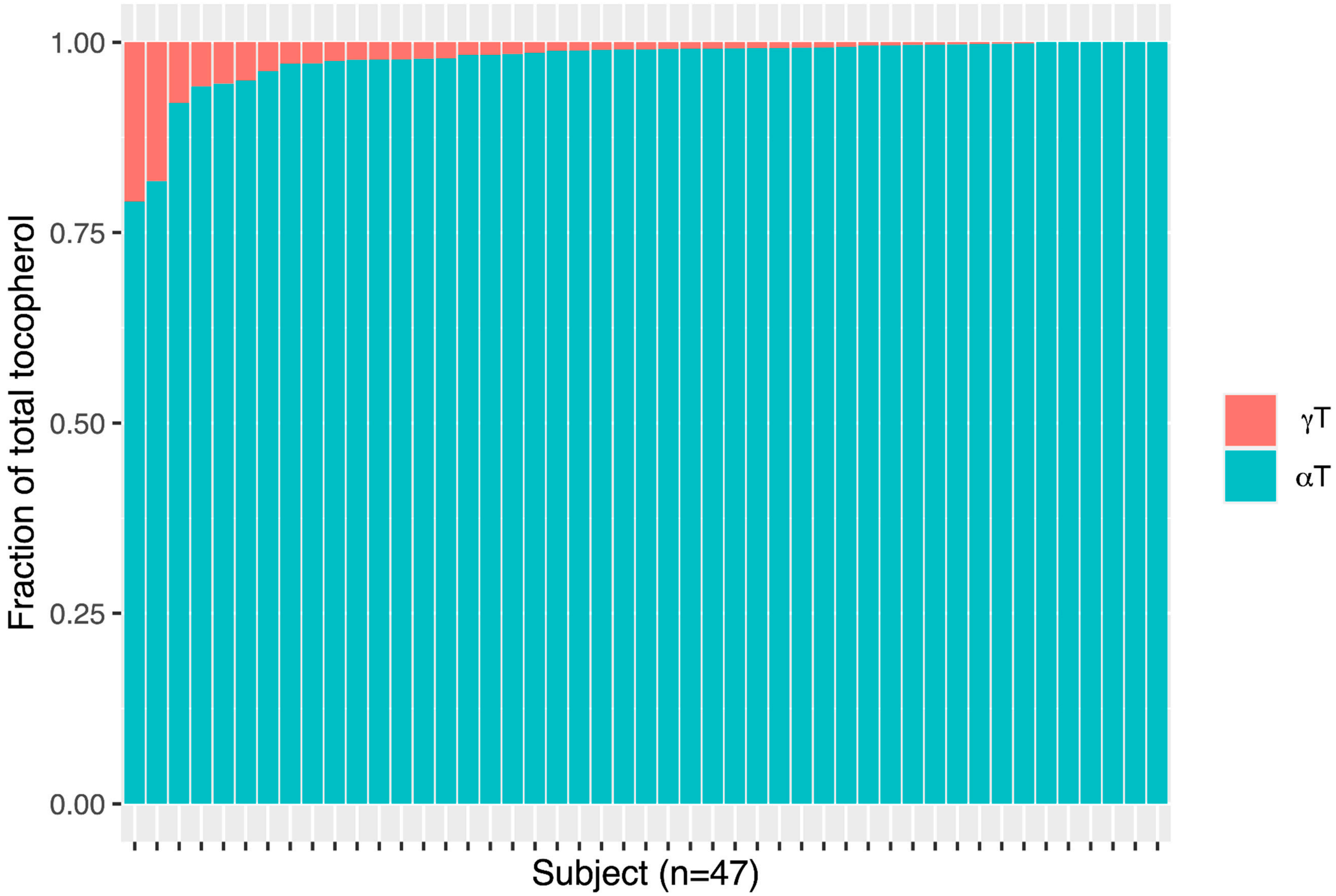
1.00
0.75
0.50
0.25
0.00
Fraction of total tocopherol
Subject (n=47)
γT
αT

(B)

Figure S1. (**A**) Tocopherol concentrations (pmol/mg tissue) in the brain of 47 subjects. Each bar represents a subject and subjects are ordered from the lowest to the highest total tocopherol concentration. αT: α-tocopherol; γT: γ-tocopherol. (**B**) Tocopherol relative concentrations (%) in the brain of 47 subjects. Each bar represents a subject and subjects are ordered from the lowest to the highest % αT. αT: α-tocopherol; γT: γ-tocopherol.

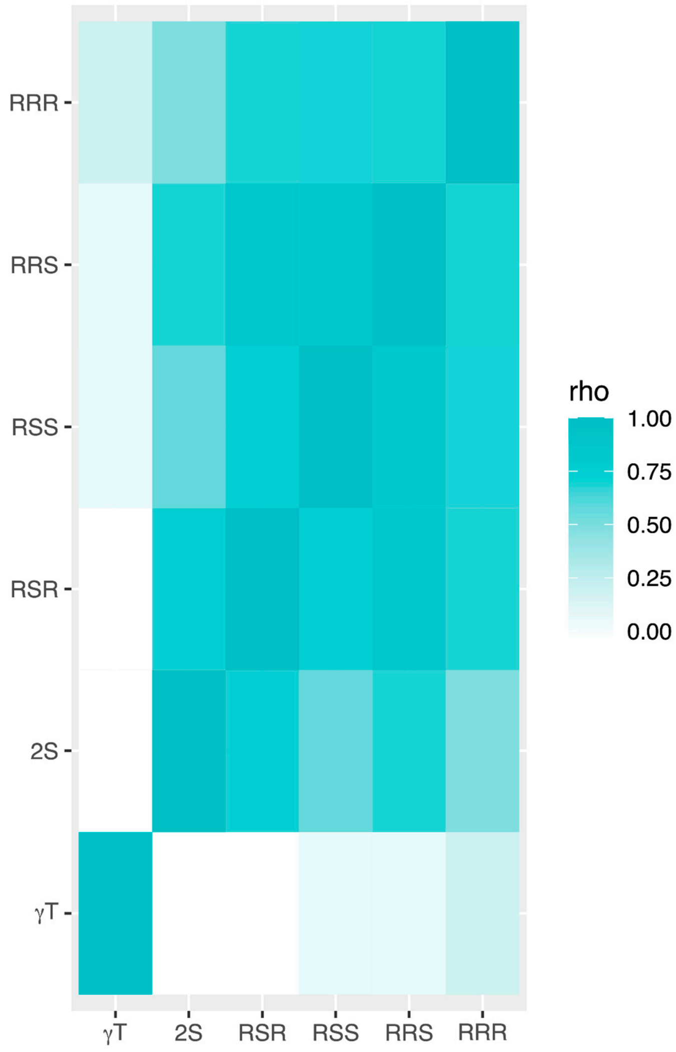

Figure S2. Spearman's rho of tocopherol absolute concentrations. *2S* includes *SSS*, *SSR*, *SRS*, *SRR*. All α-tocopherol stereoisomers were significantly and positively correlated among themselves (all $p<0.05$), but not with γ-tocopherol (γT).

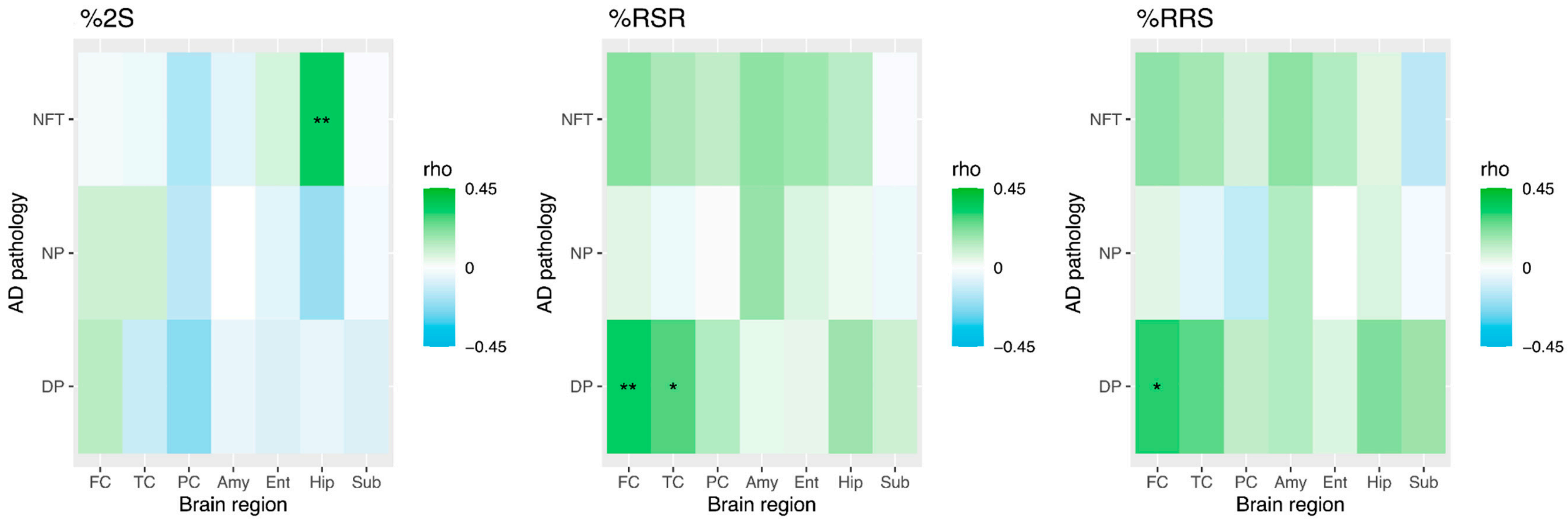

Figure S3. Correlation between *%2S*, *%RSR*, or *%RRS* and diffuse plaque (DP), neuritic plaque (NP), or neurofibrillary tangle (NFT) counts in different brain regions (n=43, excluding one double amputee and three without pathology assessment data). Partial correlation adjusting for sex, race, education, *ApoE* genotype, diabetes and hypertension. *p<0.10 and **p<0.05. FC: frontal cortex, TC: temporal cortex, PC: parietal cortex, Amy: amygdala, Ent: Entorhinal cortex, Hip: hippocampus, Sub: subiculum.

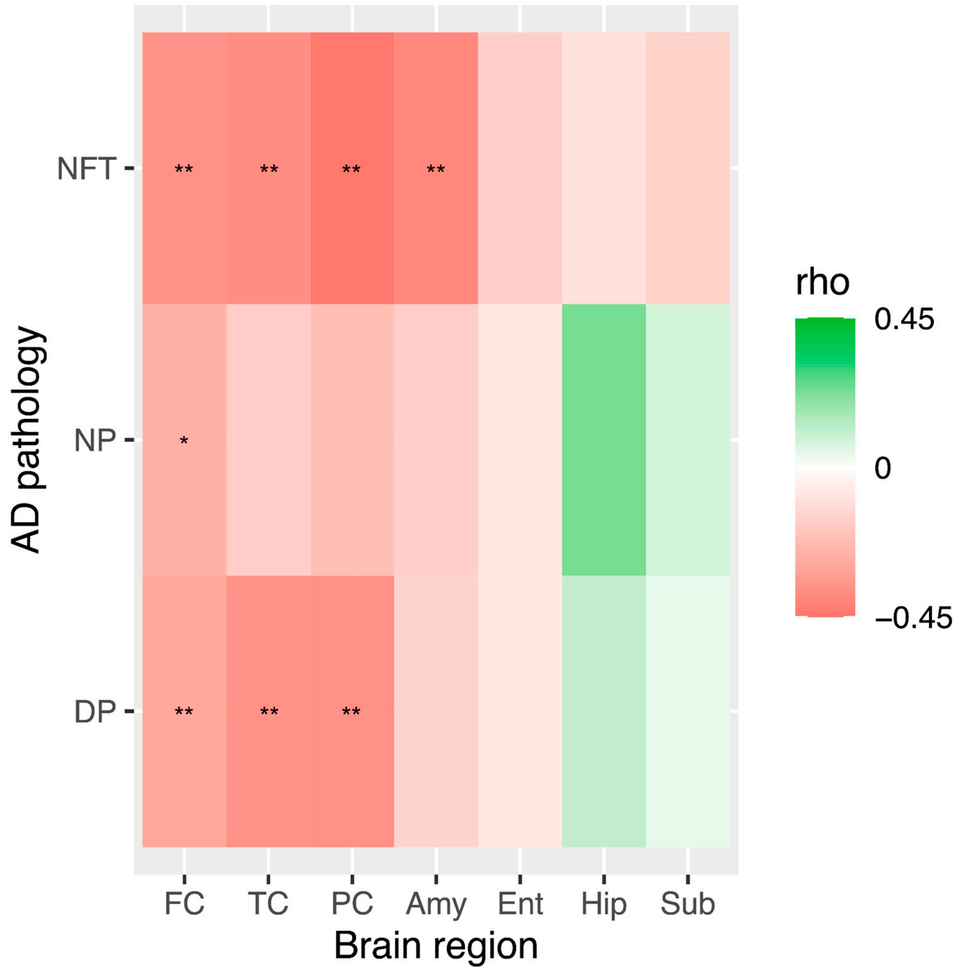

Figure S4. Correlation between body mass index and diffuse plaque (DP), neuritic plaque (NP), or neurofibrillary tangle (NFT) counts in different brain regions (n=43, excluding one double amputee and three without pathology assessment data). Partial correlation adjusting for sex, race, education, *ApoE* genotype, diabetes and hypertension. *p<0.10 and **p<0.05. FC: frontal cortex, TC: temporal cortex, PC: parietal cortex, Amy: amygdala, Ent: Entorhinal cortex, Hip: hippocampus, Sub: subiculum.
