# Supplementary material for: Associations between Brain Alpha-Tocopherol Stereoisomer Profile and Hallmarks of Brain Aging in Centenarians
Source: Antioxidants (Basel). 2024 Aug 17;13(8):997. doi: 10.3390/antiox13080997 (PMC11351880; doi:10.3390/antiox13080997)
Supplement: Supplementary file 1 [file antioxidants-13-00997-s001.zip › Supplemental_Tables.pdf]

Table S1. Correlations (Spearman’s ρ and p values) of absolute concentrations (n = 47).

	Measure	γT	2S	RSR	RSS	RRS	RRR
γT	ρ						
	p value						
2S	ρ	-0.035					
	p value	0.816					
RSR	ρ	-0.034	0.710				
	p value	0.821	<0.001				
RSS	ρ	0.065	0.566	0.730			
	p value	0.663	<0.001	<0.001			
RRS	ρ	0.055	0.671	0.863	0.817		
	p value	0.715	<0.001	<0.001	<0.001		
RRR	ρ	0.186	0.495	0.676	0.685	0.688	
	p value	0.211	<0.001	<0.001	<0.001	<0.001	

Table S2. Tocopherol concentrations (median [interquartile range]) in subjects with or without dementia. Wilcoxon rank sum test was used to compare between the two cognition groups.

Tocopherol	Absolute concentration (pmol/mg)			Relative concentration (%)*		
	GDS 1-3 No dementia (n=23)	GDS 4-7 Dementia (n=24)	p	GDS 1-3 No dementia (n=23)	GDS 4-7 Dementia (n=24)	p
γT	0.20 [0.12-0.36]	0.16 [0.06-0.26]	0.311	1.13 [0.68-3.69]	0.84 [0.33-1.79]	0.147
RRR-αT	7.36 [3.66-16.05]	10.72 [8.32-14.19]	0.249	59.83 [54.87-70.17]	63.68 [53.18-70.70]	0.975
RRS-αT	1.88 [0.71-2.96]	2.34 [1.55-3.28]	0.233	13.96 [8.56-15.99]	13.94 [10.96-17.01]	0.576
RSS-αT	1.75 [0.81-2.52]	2.03 [1.17-3.26]	0.389	13.26 [9.33-14.32]	12.98 [7.32-15.09]	0.655
RSR-αT	1.54 [0.78-2.26]	1.62 [1.22-2.55]	0.322	11.68 [8.62-12.81]	10.26 [8.23-13.84]	0.924
2S-αT	0.21 [0.11-0.38]	0.28 [0.15-0.43]	0.333	1.82 [0.84-2.58]	1.63 [1.01-2.12]	0.840

*% of αT+γT for γT, and % of total αT for αT stereoisomers.

GDS: Global Deterioration Scale

Table S3. Correlations (Spearman's ρ and p values) of tocopherol concentrations with BMI (n = 46, BMI not available for one double amputee). Relative concentrations of αT stereoisomers were calculated as % of total αT.

**Tocopherol**	**No adjustment for covariates**		**Adjusted for serum concentration***		**Adjusted for sex, race, +/- serum concentration***	
	**ρ**	**p-value**	**ρ**	**p-value**	**ρ**	**p-value**
αT+γT	-0.32	0.028	-0.32	0.030	-0.29	0.055
αT	-0.32	0.030	-0.32	0.032	-0.29	0.059
γT	0.10	0.492	0.03	0.202	0.04	0.814
2S	-0.14	0.368	NA	NA	-0.11	0.482
RSR	-0.35	0.017	NA	NA	-0.32	0.032
RSS	-0.49	<0.001	NA	NA	-0.48	<0.001
RRS	-0.36	0.013	NA	NA	-0.34	0.022
RRR	-0.20	0.192	NA	NA	-0.16	0.305
%2S	0.10	0.496	NA	NA	0.11	0.486
%RSR	-0.24	0.103	NA	NA	-0.22	0.151
%RSS	-0.45	0.002	NA	NA	-0.48	0.001
%RRS	-0.27	0.067	NA	NA	-0.27	0.073
%RRR	0.34	0.021	NA	NA	0.34	0.023

NA: not applicable

*Serum concentrations only available for total tocopherol, αT, and γT.
